# Knowledge, Attitude, and Practice of the Lebanese Community Toward COVID-19

**DOI:** 10.3389/fmed.2020.00542

**Published:** 2020-08-18

**Authors:** Souraya Domiati, Mohamad Itani, Ghida Itani

**Affiliations:** Department of Pharmacology and Therapeutics, Faculty of Pharmacy, Beirut Arab University, Beirut, Lebanon

**Keywords:** COVID-19, knowledge, attitude, pandemic, Lebanese

## Abstract

**Objectives:** Distinct measures were adopted in Lebanon to prohibit the spreading of SARS-CoV-2. These actions provide positive results only if the population chooses to be compliant.

**Aim:** Accordingly, this study aimed to reflect the Lebanese population adherence by determining their level of knowledge and practice during this pandemic.

**Method:** A cross-sectional online survey was performed in April 2020. It involved 410 volunteers from the main 5 Lebanese districts. The questionnaire was divided into 3 parts; sociodemographic, knowledge, and practice. A score was calculated out of 18 points to evaluate the knowledge of the respondents. The last 8 questions reflected the participants' precautionary methods during the pandemic. Descriptive statistics and one-way analysis of variance tests were conducted using SPSS version 20.

**Results:** The overall correct rate of the knowledge questionnaire was 75%. Survey completers of extreme age groups (under 18 and >44), elementary education level, and medical occupation displayed the least level of knowledge compared to other groups (*p* < 0.05). Most of the participants showed proactive practices to protect themselves against COVID-19. They covered their mouths (81.2%), threw the used tissues (93.7%), and washed their hands (66.6%) after sneezing or coughing. Moreover, they wore face masks if they were sick (59%) or in a crowded place (79.3%). Concerning Hydroxychloroquine, 10% claimed that they would take it if they have COVID-19 symptoms.

**Conclusion:** This survey sheds the light on the fact that one mandatory measure does not fit all the population; there must be a specialized method of prevention for each profession, age group, and area of the country to prevent the outbreak of COVID-19.

## Introduction

The Human coronavirus, which derives from the family *Coronaviradae*, includes a group of positive sensed, single-stranded RNA. These RNA viruses have the largest number of genomes ranging from 26 to 32 kilo-bases in comparison to single-stranded viruses (1). Accordingly, coronavirus has evolved by gene recombination and mutation to be subdivided into 4 categories; alpha, beta, gamma and delta (1, 2). Moreover, this virus has shown mutations with higher antigenicity, or infection potential, as a consequence of the host's development of humoral and cell-mediated immunity (3). It is one of the major pathogens that initially targets the human respiratory system (4). In fact, in December 2019, 40 cases of pneumonia were reported in Wuhan, China which etiology was soon established to be the new virus called novel coronavirus (3, 5). The severe acute respiratory syndrome coronavirus (SARS-CoV), which was the causative agent of an outbreak in 2003, and SARS-CoV-2 (the novel coronavirus), are highly similar because they have high nucleotide homology of around 77%. SARS-CoV-2 is less pathogenic, but more transmissible compared to SARS-CoV (1). SARS-CoV-2 is transmitted from one person to another by direct contact or droplets from sneezing or coughing (3).

Until the 22nd of April 2020, this outbreak resulted in 2,503,412 cases around the world whereby 171,809 lost the battle and passed away (6). The first coronavirus case in Lebanon was identified on February 21st. Eight days later, schools were locked down followed by bars, restaurants, and finally the airport. By March 15, the entire country had been on lockdown by the government due to increased case numbers (7). Even though experts at this point assumed high numbers of infected persons and mortalities, until the 25th of April, 696 cases were tested positive for coronavirus disease of 2019 (COVID-19), and 21 patients passed away (6).

As a consequence of the actions taken to prevent the spread of this outbreak, an economic crisis arose. More than 100 countries declared flight restrictions and boarder closure, causing a major drop in the number of flights per day from 150,000 to 200,000 to <100,000. The Chinese industrial production fell by 13.5% in the first 2 months of the year. According to an article published by BBC on the impact of this outbreak, 6.6 million claimed to be unemployed in the USA during April 2020 (8).

Even though everyone is at risk of developing COVID-19, yet some conditions make the patient more susceptible to the disease. Being above 65, living in a nursing home, having lung diseases, cardiovascular conditions, diabetes, liver or kidney disease, or being immune-compromised not only increases the chance of acquiring the novel coronavirus but also the complications (9).

The World Health Organization (WHO) and the Center for Disease Control and Prevention (CDC) declared that the most common signs and symptoms of this virus are fever, dry cough, and shortness of breath (9, 10). Two to 14 days and 2–10 days are the incubation periods of coronavirus which are identified by the CDC and WHO, respectively. Nevertheless, two cases were reported after being incubated for 19 and 27 days, respectively (6). WHO reported in a press conference on February 10, that a long incubation period could reflect a double exposure (6).

Up till now the golden standard of COVID-19 diagnosis is nucleic acid detection, which is obtained from throat or nose swab sampling by real-time reverse transcriptase polymerase chain reaction (11). Rapid diagnostic test (RDT) is a small and portable qualitative test that detects the presence of coronavirus antibodies; immunoglobulin G and M. The results need between 10 and 30 min to be obtained. This test was approved by food and drug administration (FDA), after the announcement of an emergency use of authorization (EUA) as a diagnostic tool (12). The WHO states that the results of this diagnostic tool can be influenced by many factors which include the time from onset of illness, the concentration of the virus in the specimen, the quality of the specimen collected, and the formulation of the reagents in the test kits. Therefore, the sensitivity of this test may vary between 34 and 80%. Moreover, this serology test doesn't indicate if the patient has an active infection. It rather indicates the presence of antibodies (13).

Unfortunately, up till today, there is no specific treatment or vaccine against the novel coronavirus. Proper symptomatic treatment along with oxygen supplementation are the major intervention for patients with severe symptoms. IDSA recommends against the use of corticosteroids in patients having pneumonia due to coronavirus. However, it supports its use when there are acute respiratory symptoms (ARDS) (14). There are several potential drug candidates including nucleoside analogs lopinavir/ritonavir, neuraminidase inhibitors, remdesivir, umifenovir, DNA synthesis inhibitors (tenofovir), tocilizumab, anti-malarial chloroquine, dexamethasone, and Chinese traditional medicine (15).

Chloroquine and Remdesivir were highly effective in the control of this virus according to a study by Lai et al. (16). A study done in France on 30 male patients showed that the antimalarial drug (hydroxychloroquine) combined with azithromycin reduced the SARS-CoV-2 load (17). The precise knowledge of hydroxychloroquine's side effects helps prevent some irreversible damages. In fact, hydroxychloroquine is not only responsible for minor side effects such as anorexia, diarrhea, and nausea, but it may also cause severe ones. Hyperpigmentation and photodynamic reaction may develop. Retinopathy will be the result of the accumulative deposition of hydroxychloroquine in the cornea (18). Concerning the heart, chloroquine, hydroxychloroquine, and azithromycin prolong the QT interval leading to a risk of arrhythmic death, especially when concomitant used (19). Consequently, the Lebanese ministry of Public health banned all pharmacies from dispensing hydroxychloroquine or chloroquine without a prescription from a specialist. Moreover, the pharmacist has to keep this prescription for tracking purposes (20). Concerning Remdesivir, an antiviral drug previously tested against Ebola virus, it did shorten the hospital stay but did not affect the mortality rate (21).

According to the RECOVERY trial, launched in March in the United Kingdom, dexamethasone decreased mortality by 20% in severe cases of COVID-19 on oxygen or ventilation. Nevertheless, dexamethasone did not affect mild infections (22).

More than 90 vaccines are being developed across the world. They work by blocking or killing the virus through exposing the body to an antigen. They rely on the virus itself, the viral vector, the nucleic acid, or the protein subunits. Other vaccines being tested are the existing ones against poliovirus or tuberculosis. Out of these vaccines, around 8 reached the safety trials while others are still being tested on animals (23).

The WHO and other health organizations declared some preventive measures. Avoid close contact, frequent handwashing with soap, and water, always carrying an alcohol-based hand sanitizer, and application of strict hygiene measures in emergency departments and hospitals. All tissues used to cover a sneeze or cough should be tossed away immediately. Finally, health care providers should utilize contact and airborne precautions. They should wear face masks, gloves, gowns, and eye protection (15).

The lack of proper awareness caused the death of 27 people in Iran not because of the virus itself but after drinking industrial alcohol believing that it is a preventive measure (24). A man and his wife died, under critical care in Arizona, after taking chloroquine in an attempt to self-medicate against SARS-CoV-2 (24). In Lebanon, the ministry of public health, in collaboration with the WHO, has issued several awareness campaigns on social media to prevent the spreading of the disease and prevent inadequate measures. Nevertheless, no study reflected the awareness of the population toward COVID-19 and its prevention. Consequently, this study was designed to evaluate the knowledge and attitude of the COVID-19 among Lebanese residents.

## Materials and Methods

### Study Design and Population

A cross-sectional anonymous survey was designed in April, 2020 targeting people living all over Lebanon. Due to quarantine, this study was conducted via a link shared on social networking platforms to limit the spreading of the disease.

### Study Tool

The survey questionnaire was designed in English and then translated to Arabic, the native language in Lebanon. Both surveys were available and the participants had the freedom to choose between the two versions.

### Pilot Study

A preliminary phase was conducted to assess the validity and reliability of the questionnaire before its use. Two experts were asked to review the questionnaire in order to make sure that it reflects the knowledge and attitude of the Lebanese population on COVID-19. Accordingly, the questionnaire was modified to meet the aim required. To check for clarity of the questionnaire, a pilot study was conducted which included 10 participants that took the survey in either language. Further modifications were done after feedback retrieval from the participants.

### Data Collection

An online open-access google form survey was created and participants from all areas of Lebanon were invited via social networking platforms to participate. Beirut, North, South, Bekaa, and Mount Lebanon were the 5 focal points. The survey link was sent to different socioeconomic levels, via WhatsApp, who were asked to spread it to their relatives and friends to overcome some limitations of the online data gathering.

### Sampling

The sample size was calculated using the online sample size “Raosoft®” calculator, assuming the Lebanese population to account for 6.825 million. The results showed that a total of 384 participants and above provides a representative sample with a 5% margin error and a 95% confidence level. The spreading of the survey link started on the 22nd of April, and this link was closed on Saturday April 25th when the number of participants exceeded the calculated representative number.

### Questionnaire

The online survey was divided into four parts that included 24 mandatory questions. The first one requiring the sociodemographic information of the participant. The second one, having 6 knowledge questions requiring multiple answers. Each right answer was given one point, and each wrong or uncertain answer was given a zero. A score out of 18 was made. The third and last part included 8 questions reflecting the attitude of the respondents.

### Statistical Analysis

The results were analyzed using Statistical Package for the Social Science (SPSS®) software version 20 (IBM, New York-USA). Categorical data were expressed as frequencies (percentages) while continuous data as means ± standard deviation (SD). The ANOVA test was used to compare means (after ensuring normality and variance homogeneity). All results were considered “statistically significant” when the *P*-value was <0.05 with a confidence interval (CI) of 95%.

### Ethical Consideration

The study was an observational one that respects the participant's confidentiality and autonomy. The participant had the choice to defer from submitting the filled form. This survey also didn't require neither names nor emails, and thus there were no traceability of the participant. Accordingly, Beirut Arab University Institution Review Board waived the approval for this study.

## Results

A total of 410 participants were included in the study. From the total participants, the age group between 25 and 44 years, female, Lebanese, and single accounted for 53.2, 58, 95.9, and 62%, respectively. Fifty-eight point five percent of the respondents live in Beirut, the capital of Lebanon. Around 80% hold a Bachelor's degree, and 32.2% work in the medical field. Concerning the participants' income per month, 31.5% reported to acquire <750,000 Lebanese pounds per month. As for the past medical history, 57.6% were in good health while 7.6%, and 4.6% had hypertension and lung diseases, respectively. Thirty-four point one percent of the participants were smokers (Table 1).

**Table 1:** Demographic data of the participants.

**Demographics**	**% (frequency)**
**Age**	
Under 18	10% (41)
18–24	25.9% (106)
25–44	53.2% (218)
45–54	8.3% (34)
55–64	1% (4)
>65	1.6% (7)
**Gender**	
Male	42% (172)
Female	58% (238)
**Nationality**	
Lebanese	95.9% (393)
Non-Lebanese	4.1% (7)
**Marital Status**	
Single	62% (254)
Married	36.1% (148)
Divorced	1.2% (5)
Widowed	0.7% (3)
**Region**	
Beirut	58.5% (240)
Mount Lebanon	29.3% (120)
South	6.1% (25)
North	5.4% (22)
Bekaa	0.7% (3)
**Highest level of education**	
None	0.2% (1)
Elementary	1.2% (5)
Secondary	15.1% (62)
University	79.8% (327)
Others	3.7% (15)
**Occupation**	
Medical	32.2% (132)
Non-medical	48.4% (182)
Unemployed	23.4% (96)
**Income/month L.L**.	
<750,000	31.5% (129)
750,000–1,500,000	18.5% (76)
1,500,000–2,250,000	18.8% (77)
2,250,000–3,000,000	11.2% (46)
3,000,000–3,750,000	5.1% (21)
>3,750,000	14.9% (61)
**Comorbidities**	
Hypertension	7.6% (31)
Diabetes	2.4% (10)
Lung disease	4.6% (18)
Others	30% (123)
None	57.6% (236)
**Smoking status**	
Cigarette	13.4% (55)
Hubble-bubble	17.5% (72)
Other	3.2% (13)
None	65.9% (270)

Most of the participants had a good knowledge of COVID-19 with a mean score of 13.51 ± 2.56 over 18. Risk factors for acquiring the novel coronavirus, as reported by the participants, were older age, cardiovascular disease, respiratory disease, diabetes, cancer, and smoking with percentages of 75.6, 73.7, 66.1, 50.5, 64.1, and 48.3%, respectively. SARS-CoV-2 was identified as a virus by 96.8% of the participants. Two to 14 days was the incubation period as acknowledged by 89% of the respondent. COVID-19 was recognized as a contagious condition by 98.1% of the participants. According to the route of transmission, 98.6 and 92.4% were positive that it can be passed via droplets and from contacting infected surfaces, respectively. On the other hand, 35.9% disagree that it is transmitted through sexual intercourse (Table 2—Part 1). Fever (97.6%), fatigue (91.5%), and shortness of breath (97.8%) were the highly acknowledged symptoms of COVID-19 (Table 2—Part 2). The participants who were under 18 years old had a mean knowledge score significantly lower than ages between 18 and 44 years. Moreover, Beirut citizens had a significantly lower score than Mount Lebanon residents but similar score with the North, Bekaa, and South areas. Concerning the level of education, the participants who only reached elementary level scored significantly the least (9.5 ± 0.22) (*p* < 0.05). Controversially, medical field workers had a significantly lower score than non-medical field workers and unemployed. On the other hand, other demographic data did not influence the knowledge of the participants (*p* > 0.05; Table 3).

**Table 2:** Knowledge on COVID-19.

**Statement or question**	**Percentage (frequency)**
**PART 1**	
**What are the risk factors for acquiring COVID-19?**	
Being an elderly patient	75.6% (310)
Being a pediatric	15.4% (63)
Having cardiovascular disease (high blood pressure, high cholesterol)	73.7% (302)
Having a respiratory disease (Asthma, COPD.)	66.1% (271)
Having diabetes	50.5% (207)
Having cancer	64.1% (263)
Being a smoker	48.3% (198)
None	1.2% (5)
I don't know	0.7% (3)
Others	0.2% (1)
**COVID-19 is caused by:**	
Virus	96.8% (397)
Bacteria	2.4% (10)
Others	0.7% (3)
**Incubation period of COVID-19 is: %(n)**	
2–14 days	89% (365)
Up to 1 month	2.4% (10)
Up to 3 months	1.0% (4)
I don't know	2.4% (10)
**Is COVID-19 a contagious disease?**	
Agree/strongly agree	98.1% (402)
Uncertain	1.5% (6)
Disagree/strongly disagree	0.5% (2)
**How is covid-19 transmitted?**	
**Through air**	
Agree/strongly agree	29% (119)
Uncertain	40% (164)
Disagree/strongly disagree	31% (127)
**Through droplets transmission from sneezing or coughing**	
Agree/strongly agree	98.6% (404)
Uncertain	0.7% (3)
Disagree/strongly disagree	0.7% (3)
**Through contacting infected surfaces**	
Agree/strongly agree	92.4% (379)
Uncertain	5.6% (23)
Disagree/strongly disagree	2% (8)
**Through sexual intercourse**	
Agree/strongly agree	29.5% (121)
Uncertain	34.6% (142)
Disagree/strongly agree	35.9% (147)
**PART 2**	
**The most common sign and symptoms of COVID-19 include:**	
**Fever**	
Agree/strongly agree	97.6% (400)
Uncertain	1.7% (7)
Disagree/strongly agree	0.7% (3)
**Fatigue**	
Agree/strongly agree	91.5% (375)
Uncertain	7.3% (30)
Disagree/strongly agree	1.2% (5)
**Shortness of breath**	
Agree/strongly agree	97.8% (401)
Uncertain	1.5% (6)
Disagree/strongly agree	0.7% (3)
**Nose bleed**	
Agree/strongly agree	21.2% (87)
Uncertain	40.2% (165)
Disagree/strongly agree	38.6% (158)
**Rash**	
Agree/strongly agree	10.5% (43)
Uncertain	37.3% (153)
Disagree/strongly agree	52.2 (214)

**Table 3:** Demographic factors associated with the knowledge.

**Demographic data**	**Mean knowledge score ± SD/18**	***P*-value**
**Age**		
Under 18	12.43 ± 2.72	–
18–24	13.84 ± 2.39	0.017
24–44	13.57 ± 2.64	0.048
45–54	13.91 ± 1.96	0.084
55–64	13.60 ± 2.07	0.923
>65	12.50 ± 2.65	1
**Gender**		
Female	13.76 ± 2.53	0.164
Male	13.16 ± 2.56	0.195
**Nationality**		
Lebanese	13.51 ± 2.55	0.129
Non-Lebanese	13.59 ± 2.83	0.687
**Marital status**		
Single	13.42 ± 2.64	–
Married	13.69 ± 2.43	0.736
Divorced	13.20 ± 1.64	0.997
Widow	12.33 ± 3.21	0.883
**Region**		
Beirut	13.13 ± 2.49	–
Mount Lebanon	14.03 ± 2.65	0.014
South	14.04 ± 2.30	0.426
North	14.32 ± 2.46	0.217
Bekaa	13.33 ± 2.52	1.000
**Education**		
Elementary	9.50 ± 0.22	–
Secondary	12.19 ± 0.29	0.051
University	13.79 ± 0.14	0.000
Others	14.47 ± 0.48	0.000
**Occupation**		
Medical	12.65 ± 2.88	–
Non-medical	13.73 ± 2.37	0.000
None	14.30 ± 2.05	0.000
**Income**		
<750,000	13.14 ± 2.73	–
750,000–1500,000	13.26 ± 2.52	0.999
1,500,000–2,250,000	13.95 ± 2.60	0.239
2,250,000–3,000,000	13.96 ± 2.53	0.425
3,000,000–3,750,000	13.71 ± 2.35	0.931
>3,750,000	13.66 ± 2.19	0.783

*The mean score was calculated from all knowledge questions*.

*ANOVA test was done followed by tukey post-hoc test*.

*P < 0.05 was considered statistically significant*.

**Figure 1:**
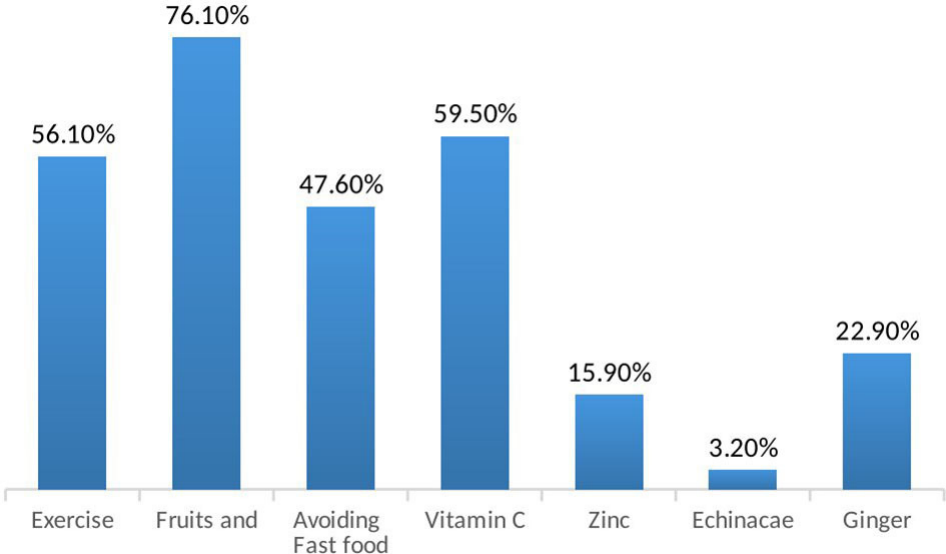
Preventive Non-pharmacological measures followed against COVID-19.

Furthermore, regarding precautions taking toward COVID-19 spreading, the overall attitude was acceptable. After sneezing or coughing, 81.2% of the respondents cover their mouths, 93.7% throw the used tissue, 93.9% turn their faces from other people, and 66.6% wash their hands. Face mask was worn by 59 and 79.3% of the participants, in case they are sick or if they are in a crowded place, respectively. Finally, 75.6% replace their face mask after a single use (Table 4). Moreover, as a measure to boost their immunity, the participants increased their fruit and vegetable (75.6%), and vitamin C (59.5%) intakes. More than half of the participants (56.1%) started conducting light exercises. Other measures taken were avoiding take away food (47.6%), consuming ginger (22.9%), Echinacea (3.2%), and zinc (15.9%) as supplements (Figure 1).

**Table 4:** Attitude toward COVID-19.

	**Always % (*n*)**	**Sometimes % (*n*)**	**Never % (*n*)**
**After sneezing/coughing do you**			
Cover your mouth	81.2% (333)	17.1% (70)	1.7% (7)
Throw the used tissue	93.7% (384)	4.9% (20)	1.5% (6)
Turn your face	93.9% (385)	3.9% (16)	2.2% (9)
Wash your hands	66.6% (273)	30.5% (125)	2.9% (12)
**Do you wear a face mask if you are**			
Sick	59% (242)	27.3(112)	13.7% (56)
In a crowded place	79.3% (325)	14.6% (60)	6.1% (25)
**Do you replace the face mask after**			
Single use	75.6% (310)	19% (78)	5.4% (22)

Referring to studies on hydroxychloroquine, only 10% agreed to take hydroxychloroquine if signs and symptoms of COVID-19 were present. The most serious side effects of the anti-malarial drug recognized by the participants were electrical disturbances of the heart (38%) and eye damage (18.8%). On the other hand, 50% did not recognize any side effect of hydroxychloroquine (Tables 5, 6).

**Table 5:** Attitude of the participants toward Hydroxychloroquine use in COVID-19 infection.

**In case of having COVID-19 symptoms, would you take Hydroxychloroquine if it was available at home?**	**% (frequency)**
Yes	10% (41)
No	70.5% (289)
Maybe	19.5% (80)

**Table 6:** Knowledge of the participants on the side effects of Hydroxychloroquine.

**Most serious side effects of Hydroxychloroquine**	**% (frequency)**
Eye damage	18.8% (77)
Electric Disturbances of the heart	38% (156)
Headache	11.7% (48)
Nausea	12.7% (52)
Abdominal pain	10.7% (44)
I don't know	50% (205)
Others	1.2% (5)

**Figure 2:**
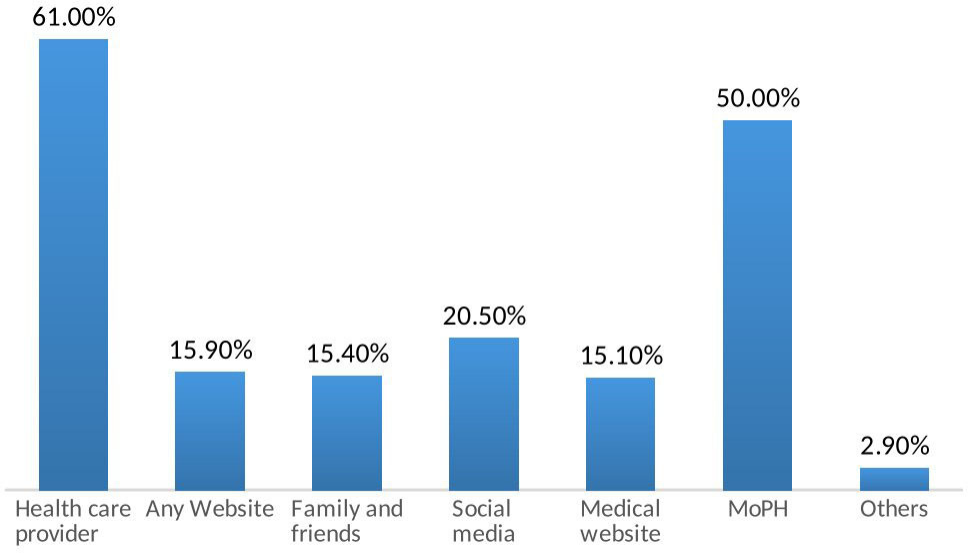
Medical resources for inquiries on COVID-19.

For medical inquiry, the survey participants prefer to seek the advice of a health care provider (60%) or the Ministry of Public Health hotline (50%). Nevertheless, 20% seek medical information from social media platforms (Figure 2). In the presence of COVID-19 symptoms, 52.7% of the participants consider calling the Lebanese Red Cross to be transferred to the assigned hospitals, 25% visit any hospital, 10.5% seek help from their health care provider (physician, pharmacist.), and 11.2% self-medicate themselves without seeking medical advice (Table 7).

**Table 7:** Attitude of the participants in the presence of COVID-19 symptoms.

**In case I have COVID-19 symptoms I would**	**% (frequency)**
Seek emergency at any hospital	25.6% (105)
Call the Lebanese Red Cross to be taken to COVID-19 assigned hospitals	52.7% (216)
Seek health care providers (pharmacist, physician.)	10.5% (43)
Don't refer to any and self-medicate	11.2% (46)

## Discussion

COVID-19 represents a global health threat that boosts all local and international organizations to take preventive measures. In general, measures should encompass the source of the infection, its transmission route, and the susceptible population. Accordingly, knowledge of these vital elements is a necessity (25, 26). In the current study, the respondents displayed a decent knowledge regarding risk factors, etiology, and route of transmission, incubation period, and signs and symptoms of COVID 19. The mean knowledge score was 13.51 ± 2.56 over 18 which can be attributed to the fact that nearly 80% of the participants had a university degree. Moreover, the main primary sources of COVID-19 information for the survey participants were health care providers (61%), and the Lebanese Ministry of Public Health (50%). In fact, the Lebanese authorities have released a new website for all information regarding the COVID-19. Moreover, television channels have broadcasted preventive measures (27). Nevertheless, despite all the effort taken by the Ministry of Health through schools, under 18 were the least informed on this condition followed by the elderly. In fact, age groups >50 years had similar results to Chinese and Egyptian residents (28–30). An alarming finding showed that Lebanese health care providers scored lower than non-healthcare providers. According to a survey on the perception and knowledge of health care workers, this discrepancy can be explained by the fact that some are experts in other domains than infectious diseases (31). This finding imposes that awareness should target all categories of citizens. Two distinct websites should be created; one for the health care providers and one for the non-healthcare providers. Television channels should broadcast animated recommendations targeting the children. Areas of low knowledge should be targeted by posting on the road billboards and phone message notifications.

Knowledge is a requirement for establishing prevention beliefs, developing positive attitudes, and encouraging positive behaviors toward the disease (32). This was reflected in the practice of most of the participants in the survey. Nevertheless, even though 98.6% of the participants agreed that SARS-CoV-2 is transmitted through sneezing or coughing droplets, 17.1 and 1.7% sometimes and never cover their mouth after sneezing or coughing in public places, respectively. Moreover, 14.6% sometimes wear a mask in crowded places and 6.1% never did. Being sick also did not trigger wearing a mask in 13.7% of the respondents. Consequently, the virus can be transmitted easily by this minority since it is highly contagious.

Even though the U.S. National Institutes of Health does not recommend the use of any agent as prophylaxis against COVID-19 (33), 76.1% increased their consumptions of fruits and vegetables, 59.5% consumed vitamin C supplements, 56.1% did exercise on a daily basis, and 47.6% avoided fast food in an attempt to increase their immunity and consequently decrease the risk of acquiring the virus. These lifestyle modifications are a necessity to avoid most diseases including diabetes and cardiovascular diseases.

According to the Ministry of Health, patients with symptoms should contact the ministry through the hotline or call the Red Cross to be transferred to the assigned hospital. Moreover, the Lebanese Order of Pharmacists stated that anyone with fever, shortness of breath, or cough, or had been in contact with COVID-19 patients, or had been outside the country is not allowed to enter the pharmacies and has to refer to the assigned hospitals by the Ministry of Health. With all these restrictions, 10.5% still would seek pharmacists or physician clinics in case of having COVID-19 symptoms which puts the health care provider along with staff, waiting patients, and possible pediatrics at risk of developing the disease. Furthermore, 11.2% will choose to self-medicate instead of medical referral. This is alarming, especially that 10% will have a tendency to take hydroxychloroquine, if it is available at home. The National Institutes of Health stated that there isn't enough data to recommend or ban the use of hydroxychloroquine or chloroquine for the treatment of COVID-19 (33). In addition, hydroxychloroquine may cause life-threatening side effects, such as fatal cardiac arrhythmias, which is known by only 38% of the questioned participants.

## Conclusion and Recommendation

This study showed that there is a fair knowledge and positive attitude toward COVID-19. However, more awareness campaigns should be conducted as new cases were reported. The personnel in charge should develop a plan in a way that limits the transmission of this disease once quarantine is lifted. Face masks, should not be put by choice, enforcing the mandatory wearing of a face mask in public should be a must. Enforcing laws, including the allowance of a limited number of personnel in a supermarket and shops based on its area, could also provide the required social distancing and thus limits the spread of the virus. Finally, proper awareness should not only be restricted to social media platforms nor Televisions; but also the Ministry of Public Health should come up with a focus group to target both educated and uneducated, extreme age groups, and poor sanitary areas of the country.

## Data Availability Statement

The datasets presented in this article are not readily available because it contains some confidential information. Requests to access the datasets should be directed to t.domyati@bau.edu.lb.

## Ethics Statement

Ethical review and approval was not required for the study on human participants in accordance with the local legislation and institutional requirements. The patients/participants provided their written informed consent to participate in this study.

## Author Contributions

The idea of the research was suggested by GI. This study design was elaborated by all authors. SD did the data analysis. All authors were involved in the writing and revising processes.

## Conflict of Interest

The authors declare that the research was conducted in the absence of any commercial or financial relationships that could be construed as a potential conflict of interest.
